# Protective Effects of Anwulignan against HCl/Ethanol-Induced Acute Gastric Ulcer in Mice

**DOI:** 10.1155/2021/9998982

**Published:** 2021-07-14

**Authors:** Jiawei Liu, Huijiao Lin, Liwei Yuan, Dan Wang, Chunmei Wang, Jinghui Sun, Chengyi Zhang, Jianguang Chen, He Li, Shu Jing

**Affiliations:** ^1^Department of Pharmacology, College of Pharmacy, Beihua University, Jilin 132013, China; ^2^Department of Pathology, College of Medicine, Beihua University, Jilin 132013, China; ^3^Department of Digestive Surgery, Affiliated Hospital of Beihua University, Jilin 132013, China

## Abstract

Gastric ulcer is one of the most common gastrointestinal diseases. Anwulignan (AN) is a major active component of *Schisandra sphenanthera* Rehd. This study was designed to evaluate the protective effect of AN against the acute gastric ulcer induced by HCl/ethanol in mice. The mice were given HCl/ethanol by gavage to establish an acute gastric ulcer model. Then, the serum and gastric tissue samples were taken for biochemical analyses. The results showed that the pretreatment with AN could significantly reduce the gastric ulcer index (GUI) and increase the ulcer inhibition rate, indicating that AN can protect against gastric ulcers. AN showed its antioxidant roles by decreasing the content of reactive oxygen species (ROS), malondialdehyde (MDA), and 8-hydroxydeoxyguanosine (8-OHdG) and increasing the activity of superoxide dismutase (SOD), catalase (CAT), and glutathione peroxidase (GSH-Px) and anti-inflammatory roles by decreasing the content of tumor necrosis factor-*α* (TNF-*α*), interleukin-6 (IL-6), interleukin-1*β* (IL-1*β*), and myeloperoxidase (MPO) and increasing the content of interleukin-2 (IL-2), interleukin-4 (IL-4), interleukin-10 (IL-10), prostaglandin E2 (PGE2), and nitric oxide (NO) in both serum and gastric tissue. Furthermore, AN also activated the NRF2/ARE signaling pathway and inhibited the MAPK/NF-*κ*B signaling pathway. AN improves the acute gastric ulcer induced by HCl/ethanol in mice, which may be mainly through its antioxidant capacity and anti-inflammatory effect.

## 1. Introduction

Gastric ulcer often occurs in the gastric horn, antrum, cardia, and hiatal hernia, and about 10% of the world's population suffer from this disease and 1% of them are more likely to deteriorate into cancer; therefore, gastric ulcer has been classified as a precancerous disease by WHO [1, 2]. Gastric ulcer is generally induced by smoking, drinking, poor diet, stress, and the abuse of nonsteroidal anti-inflammatory drugs (NSAIDs) [3, 4]. Among them, excessive drinking is the most common cause, and the underlying mechanisms may be related to oxidation and inflammation [5–8]. Therefore, antioxidation and anti-inflammation are supposed to be effective strategies to prevent and treat gastric ulcers. The oxidative injury and inflammatory reaction in the tissue of gastric ulcer induced by alcohol will be further aggravated by gastric acid [9]. Accordingly, a modified gastric ulcer model induced by HCl/ethanol has been widely used for the study on the pathogenesis of gastric ulcer and the evaluation of the efficacy of antiulcer drugs [10].

Drugs for the treatment of gastric ulcer at present are mainly chemosynthetic drugs, including H_2_ receptor blockers, proton pump inhibitors, and gastric mucosal protective agents. However, these drugs, though effective, tend to cause severe adverse reactions, such as hepatitis, diarrhea, and headache [11]. Therefore, many extracts or active components from natural plants have been focused on as alternatives to these drugs [12, 13]. Herbal medicine has been used with a long history in clinical practice and proved effective in the treatment of many diseases [14]. Schisandra, the dried and mature fruit of *Schizandra sphenanthera* Rehd., is a commonly used herb in China, South Korea, and Russia [15]. It possesses the activities of astringency, nourishing, liver protection, anticancer, and antioxidation and has been considered an ideal resource for the development of drugs and healthy foods [16, 17]. With more and more understanding of its functions, Schisandra is widely used in medicine, health care products, food, and beverage industries [18]. Anwulignan (AN) used in this study is the representative active monomer in Schisandra lignans. Our previous work showed that AN had strong antioxidant and anti-inflammatory activities in both the fatigue model [19] and D-galactose-induced aging model of mice [20]. However, to the best of our knowledge, there is no report on the protective effect of AN against gastric ulcer until now.

In this study, by using HCl/ethanol-induced acute gastric ulcer in mice, we observed the protective effect of AN against gastric ulcer and also explored the related mechanism based on its antioxidation and anti-inflammation.

## 2. Materials and Methods

### 2.1. Drugs and Reagents

The study used the following: AN, purity of 99.76%, Sichuan Weikeqi Biological Technology Co., Ltd., Chengdu, China; SOD, MDA, CAT, GSH-Px, and ROS detection kits (Nanjing Jiancheng Bioengineering Research Institute, Nanjing, China); 8-OHdG, NO, PGE2, and MPO detection kits (Shanghai Enzyme-Linked Biotechnology Co., Ltd., Shanghai, China); IL-2, IL-4, IL-6, IL-1*β*, IL-10, and TNF-*α* detection kits (ABclonal Technology Co., Ltd., Wuhan, China); nuclear factor erythroid 2-related factor 2 (NRF2), phospho-NRF2 (S40), Kelch-like ECH-associated protein 1 (Keap1), heme oxygenase 1 (HO-1), P-65, P-P65, I*κ*B*α*, P-I*κ*B*α*, P-38, P-P38, JNK, P-JNK, ERK1/2, P-ERK1/2, and GAPDH antibodies (ABclonal Technology Co., Ltd., Wuhan, China); and ranitidine (RAN, Yunpeng Pharmaceutical Group Co., Ltd., Shanxi, China). All the other chemicals were purchased from Beijing Chemical Plant Co., Ltd. (Beijing, China).

### 2.2. Animals and Experimental Protocol

Fifty-five adult male ICR mice, 6–8 weeks and 18–22 g, were purchased from Changchun Yisi Experimental Animal Co., Ltd. (Changchun, China), with the quality certificate of SCXK (Ji)-2018-0007. The experimental protocols involving animals were reviewed and approved by the Institutional Animal Care and Use Committee (IACUC) of Beihua University (approval no. CPBHU IACUC2019-018) and carried out according to the established animal research guidelines. In a temperature- and humidity-controlled environment, mice were kept in a 12-hour light/dark cycle and under specific pathogen-free conditions and had free access to food and water.

**Figure 1:**
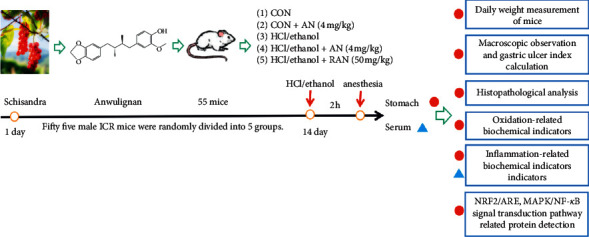
Experimental protocol.

The experimental protocol is shown in Figure 1. Fifty-five male ICR mice were randomly divided into 5 groups: (1) control (CON) group; (2) CON + AN group; (3) HCl/ethanol group; (4) HCl/ethanol + AN group; and (5) HCl/ethanol + RAN (ranitidine) group. Mice in CON + AN group and HCl/ethanol + AN group were administered with AN, those in HCl/ethanol + RAN group with RAN, and those in CON and HCl/ethanol group with an equal volume of solvent by gavage once a day for 14 days. According to our previous study and preliminary experiment, AN at the dose of 4 mg/kg showed good antioxidant and anti-inflammatory activity [19, 20], so we chose this dose in the present study. In addition, ranitidine at a dose of 50 mg/kg was used as a positive control [21–23]. The mice were fasted for 24 hours and were not allowed to drink for 4 hours before the experiment. One hour after the last administration of the above treatments, the mice were given HCl/ethanol (0.1 ml/10 g) by gavage to establish an acute gastric ulcer model. Two hours later, the mice were anesthetized by the intraperitoneal injection of pentobarbital for euthanasia, and then the blood and gastric tissue were taken for the corresponding analyses.

### 2.3. Establishment of HCl/Ethanol-Induced Gastric Ulcer Model

Except for those in the CON group and CON + AN group, the mice in the other groups were given 0.1 ml HCl/ethanol per 10 g BW and 60% 150 mmol HCl by gavage to establish an acute gastric ulcer model of mice [24].

### 2.4. Measurement of the Body Weight of Mice

During the experiment, the body weights of mice in all groups were measured every day, and the curves of body weight changes were drawn.

### 2.5. Gross Observation of Gastric Mucosa and Assessment of Gastric Tissue Injury

The excised stomach was opened along the greater curvature and rinsed completely with cold saline for 30 minutes. Then, the stomachs were blotted dry with filter paper, spread out, and photographed. Gastric ulcer index (GUI), a classic index to evaluate the degree of gastric mucosal injury, was calculated as 0 = no injury, 1 = minor hemorrhagic lesion, 2 = lesion < 2 mm (the length of ulcer or erosion), 3 = lesion from 2 to 3 mm, 4 = lesion from 3 to 4 mm, and 5 = lesion > 4 mm; the score was doubled when the erosion width was more than 1 mm [25–27]. Ulcer inhibition rate is another important index to evaluate the degree of gastric mucosal injury, which was calculated as inhibition ratio (%) = [(GUI of HCl/ethanol group − GUI of treated group)/(GUI of HCl/ethanol group)] × 100.

### 2.6. Histopathological Examination

After the gross examination, the gastric tissues were fixed with 10% formalin solution for more than 48 hours, then dehydrated in gradient alcohol and embedded with paraffin, and sliced into 4 *μ*m thick. Hematoxylin and eosin (H&E) staining was used and the pathological changes of the gastric tissue were observed with an optical microscope.

### 2.7. Detection of Biochemical Indexes Related to Oxidative Stress

The gastric homogenate was prepared by mixing the gastric tissue of mice with cold PBS at the ratio of 1 to 9. The homogenate was centrifuged at 3500 r/min and 4°C for 15 min. The supernatant was collected and frozen at −80°C. The activities of SOD, CAT, and GSH-Px, as well as the contents of ROS, MDA, and 8-OHdG, were detected according to the methods provided by the kit manufacturers.

### 2.8. Detection of Biochemical Indexes Related to Inflammatory Reaction

Two hours after the model was established with HCl/ethanol, the blood of mice was collected from the orbit of mice and centrifuged at 4°C 3500 r/min for 15 min, and then the supernatant was collected and frozen at −80°C. The levels of TNF-*α*, IL-6, IL-1*β*, IL-2, IL-4, IL-10, MPO, PGE2, and NO in both serum and gastric tissue were detected by using enzyme-linked immunosorbent assay (ELISA), and the specific methods were referred to the instructions of the kits.

### 2.9. Western Blot Analysis of NRF2/ARE and MAPK/NF-*κ*B Signaling Pathway Proteins in the Gastric Tissue

Gastric tissues of 3 mice in each group were treated with the lysis buffer (containing protease inhibitor and phosphatase inhibitor) on ice for 1 h and then centrifuged at 12,000 r/min for 10 min, and their supernatants were collected. The protein concentration in the gastric tissue was determined by BCA method. P-NRF2, NRF2, Keap1, HO-1, P-65, P-P65, I*κ*B*α*, P-I*κ*B*α*, P-38, P-P38, JNK, P-JNK, ERK1/2, P-ERK1/2, and GAPHD proteins were separated by sodium dodecyl sulphate-polyacrylamide gel electrophoresis (SDS-PAGE) and transferred onto polyvinylidene fluoride (PVDF) membranes for 2 h. The membranes were rinsed with Tris buffer (TBST) for 5 min and then blocked with the TBST buffer containing 5% skimmed milk powder for 2 h at room temperature. Then, the blocking buffer was discarded, and the antibodies of P-NRF2 (1 : 1000), NRF2 (1 : 1000), Keap1 (1 : 1000), HO-1 (1 : 1000), P-65 (1 : 1000), P-P65 (1 : 1000), I*κ*B*α* (1 : 1000), P-I*κ*B*α* (1 : 1000), P-38 (1 : 1000), P-P38 (1 : 1000), JNK (1 : 1000), P-JNK (1 : 1000), ERK1/2 (1 : 1000), and P-ERK1/2 (1 : 1000) were added onto the membranes. The membranes were incubated at 4°C overnight and then washed with TBST (3 × 10 min). The second antibody (1 : 5000) was added onto the membranes, and the membranes were incubated at room temperature for 1 h. Then, the membranes were washed with TBST again (3 × 10 min) and developed with enhanced electrogenerated chemiluminescence (ECL) developer.

### 2.10. Statistical Analysis

The data were expressed as mean ± standard deviation (mean ± SD) while ulcer index is expressed in terms of median (min–max). SPSS 20.0 statistical software was used for the statistical analysis of one-way ANOVA. Differences among the experimental groups were determined by one-way ANOVA followed by the *t*-test or Kruskal–Wallis analysis of variance followed by the Mann–Whitney *U* test for multiple comparisons. *P* < 0.05 was considered to be statistically significant.

## 3. Results

### 3.1. No Effect of AN on the Body Weight of Mice

**Figure 2:**
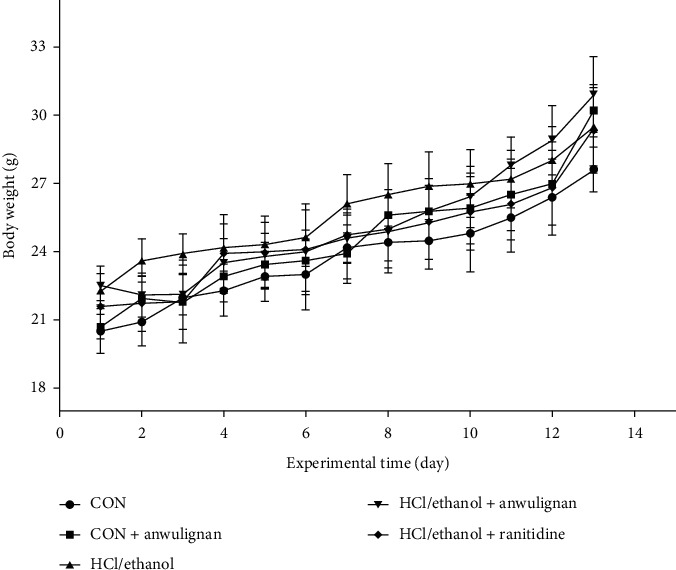
Body weight changes of mice in different groups. The data are shown as mean ± SD, *n* = 11.

As shown in Figure 2, the body weight of mice in each group increased, but without significant difference among groups.

### 3.2. Protective Effect of AN against the Gastric Ulcer Induced by HCl/Ethanol

**Figure 3:**
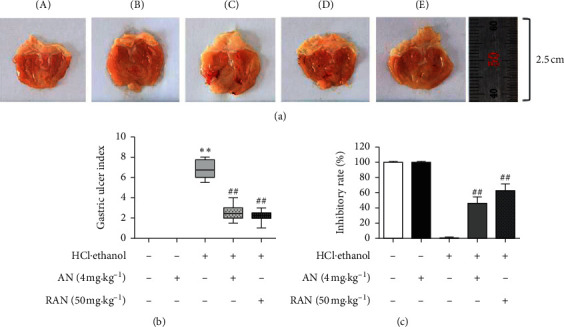
Protective effect of AN against the gastric ulcer induced by HCl/ethanol in mice. (a) Gross observation of gastric mucosa. (b) GUI. (c) Ulcer inhibition rate. A, CON group; B, CON + AN group; C, HCl/ethanol group; D, HCl/ethanol + AN group; and E, HCl/ethanol + RAN group. GUI is expressed as median (min–max). Ulcer inhibition rates are shown as the mean ± SD, *n* = 8. ^∗∗^Compared with the CON group, *P* < 0.01; ^##^compared with HCl/ethanol group, *P* < 0.01.

As shown in Figure 3(a), the gastric mucosa of mice in the CON group and CON + AN group was smooth and light pink, but without ulcer and hyperemia; in contrast, in the groups treated with HCl/ethanol, the gastric mucosa showed severe damage with dotted bleeding points and erosion; AN and RAN could significantly improve the damage of gastric tissue and reduce the numbers of ulcer sites and bleeding points.

GUI and ulcer inhibition rate are the direct indicators to evaluate the degree of gastric injury. As shown in Figures 3(b) and 3(c), the GUI in the HCl/ethanol group was 6.75 (5.5–8.0) significantly higher than that in the CON group (*P* < 0.01), the GUI in the AN- and RAN-pretreated groups was 2.5 (1.5–4.0) and 2.25 (1.0–3.0) significantly lower than that in the HCl/ethanol group (*P* < 0.05), and the ulcer inhibition rate in AN- and RAN-pretreated groups was 46% and 63%, respectively, suggesting that AN may have a protective effect against the gastric ulcer induced by HCl/ethanol in mice.

### 3.3. Improvement of AN on the Pathological Changes of Gastric Ulcer Induced by HCl/Ethanol

**Figure 4:**
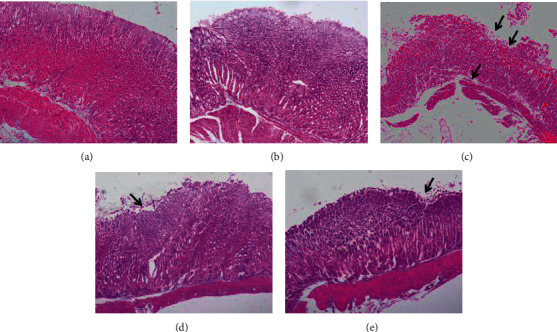
Effects of AN on the pathology of gastric ulcer induced by HCl/ethanol in mice. The gastric tissue was fixed with 10% formaldehyde, embedded in paraffin, sliced with a microtome, and stained with hematoxylin and eosin (H&E, 200x). (a) CON group; (b) CON + AN group; (c) HCl/ethanol group; (d) HCl/ethanol + AN group; and (e) HCl/ethanol + RAN group. Arrows indicate endothelial erosion and ulceration.

The histopathological examination (Figure 4) showed that the structure of gastric mucosa was continuous and intact, the epithelial cells are normal, and there were no obvious congestion and edema in the gastric mucosa of mice in the CON and CON + AN groups, while HCl/ethanol caused extensive damage to the gastric tissue, including the defect of mucosal epithelial cells, the decrease of mucosal glands, and the infiltration of submucosal inflammatory cells; after pretreatment with AN, the defect of gastric epithelial cells was improved, the hemorrhagic injury and inflammatory cell infiltration were alleviated, and there were no obvious congestion and edema in the gastric mucosa, which were similar to those caused by the treatment with RAN.

### 3.4. Suppression of AN on Oxidative Stress-Related Biochemical Indicators in the Gastric Tissue of Mice Treated with HCl/Ethanol

**Figure 5:**
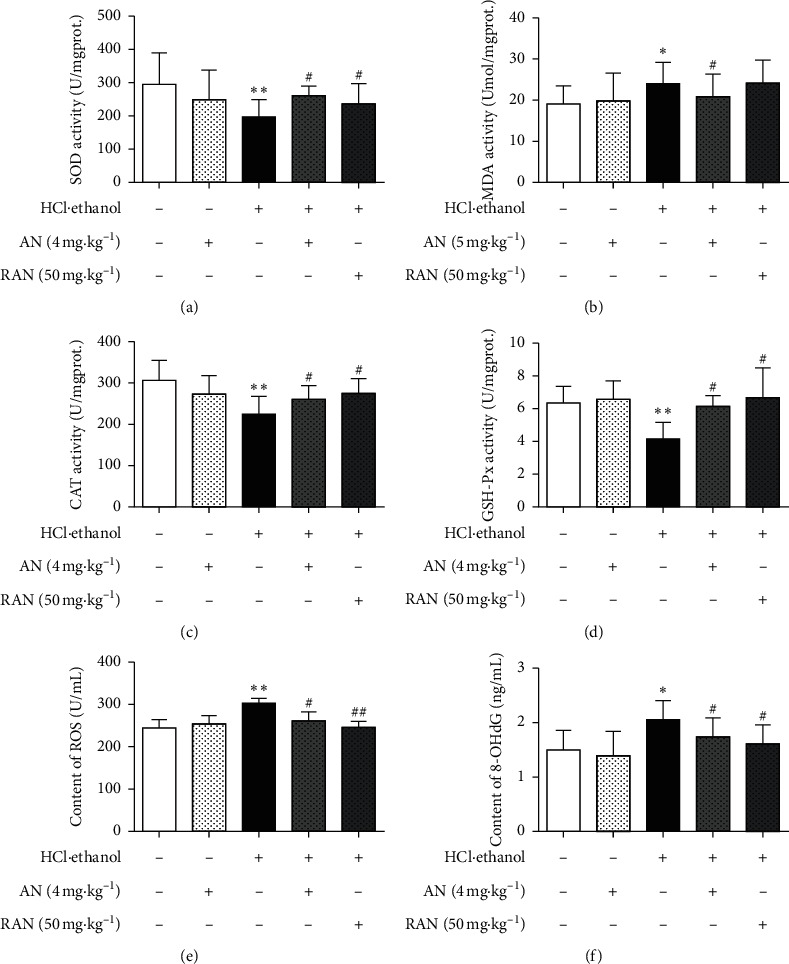
Effects of AN on oxidative stress-related biochemical indicators in the gastric tissue of mice treated with HCl/ethanol. (a) SOD activity; (b) MDA content; (c) CAT activity; (d) GSH-Px activity; (e) ROS content; and (f) 8-OHdG content. The data are presented as mean ± SD (*n* = 8). ^∗^Compared with the CON group, *P* < 0.05; ^*∗∗*^compared with the CON group, *P* < 0.01; ^#^compared with the HCl/ethanol group, *P* < 0.05; ^##^compared with the HCl/ethanol group, *P* < 0.01.

Oxidative stress plays an important role in the acute gastric ulcer induced by HCl/ethanol in mice. In order to observe the antiulcer effect of AN on oxidative stress, the activities of SOD, CAT, and GSH-Px and the contents of ROS, MDA, and 8-OHdG in the gastric tissue were measured. As shown in Figure 5, the activities of SOD, CAT, and GSH-Px in the gastric tissue of mice in the HCl/ethanol group were significantly lower than those in the CON group (*P* < 0.05 or *P* < 0.01). However, the contents of ROS, MDA, and 8-OHdG in the gastric tissue of mice in the HCl/ethanol group were significantly higher than those in the CON group (*P* < 0.05 or *P* < 0.01) and the activities of SOD, CAT, and GSH-Px in the gastric tissue of mice in the HCl/ethanol + AN and HCl/ethanol + RAN groups were significantly higher than those in HCl/ethanol group (*P* < 0.05 or *P* < 0.01), while the contents of ROS, MDA, and 8-OHdG in the gastric tissue of mice in HCl/ethanol + AN and HCl/ethanol + RAN groups were significantly lower than those in the HCl/ethanol group (*P* < 0.05 or *P* < 0.01); there was no significant difference in the activity of SOD, CAT, and GSH-Px and the content of ROS, MDA, and 8-OHdG between the CON group and CON + AN group, indicating that AN could improve the antioxidant capacity and alleviate the oxidative damage of gastric tissue induced by HCl/ethanol.

### 3.5. Inhibition of AN on Inflammation-Related Factors in the Serum and Gastric Tissue of Mice Treated with HCl/Ethanol

**Figure 6:**
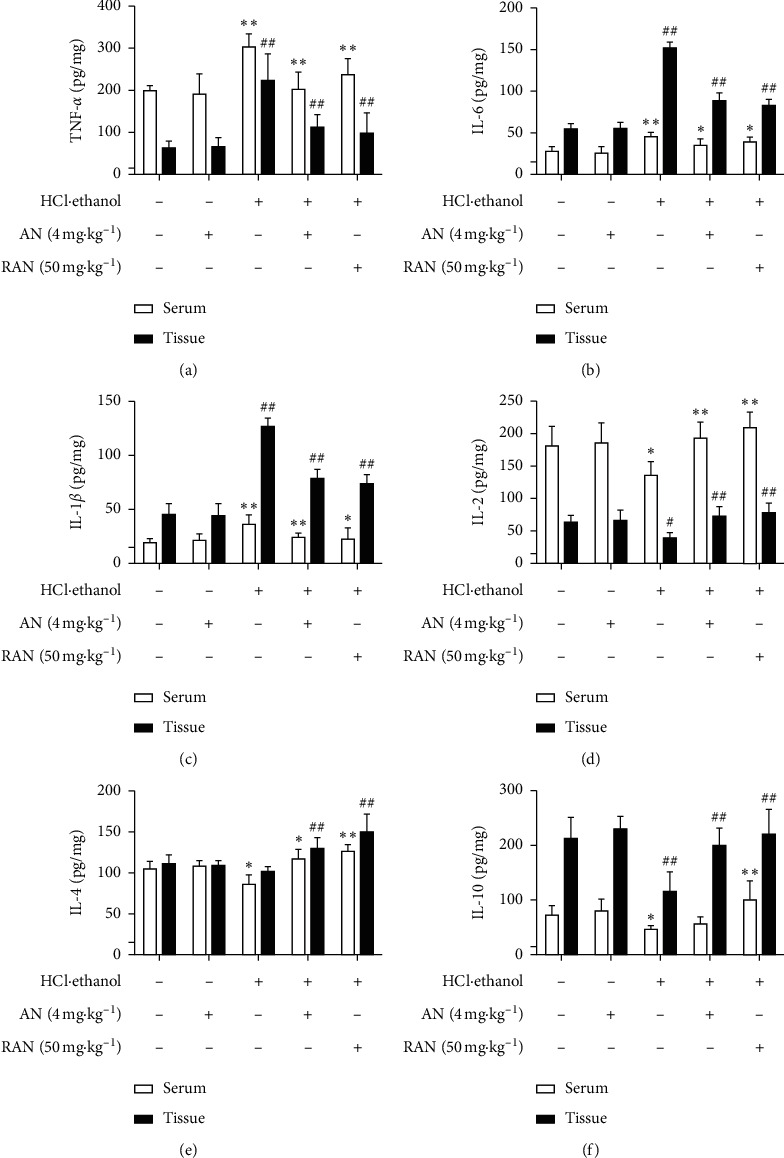
Effects of AN on inflammation-related factors in the serum and gastric tissue of mice treated with HCl/ethanol. (a) Content of TNF-*α*. (b) Content of IL-6. (c) Content of IL-1*β*. (d) Content of IL-2. (e) Content of IL-4. (f) Content of IL-10. The data are presented as mean ± SD (*n* = 8). ^∗^Compared with CON group, *P* < 0.05; ^∗∗^compared with CON group, *P* < 0.01; ^#^compared with HCl/ethanol group, *P* < 0.05; ^##^compared with HCl/ethanol group, *P* < 0.01.

Proinflammatory factors, such as TNF-*α*, IL-6, and IL-1*β*, and anti-inflammatory factors, such as IL-2, IL-4, and IL-10, are of great significance in the inflammatory response [28]. As shown in Figure 6, compared with those in the CON group, the contents of proinflammatory cytokines TNF-*α*, IL-6, and IL-1*β* were significantly increased (*P* < 0.05 or *P* < 0.01), and the contents of anti-inflammatory cytokines IL-2, IL-4, and IL-10 were significantly decreased (*P* < 0.05 or *P* < 0.01) in the serum and gastric tissue of mice in the HCl/ethanol group, while the pretreatment with AN and RAN could significantly reduce the contents of TNF-*α*, IL-6, and IL-1*β* (*P* < 0.05 or *P* < 0.01), and increase the contents of IL-2, IL-4, and IL-10 (*P* < 0.05 or *P* < 0.01) in the serum and gastric tissue of mice treated with HCl/ethanol.

**Figure 7:**
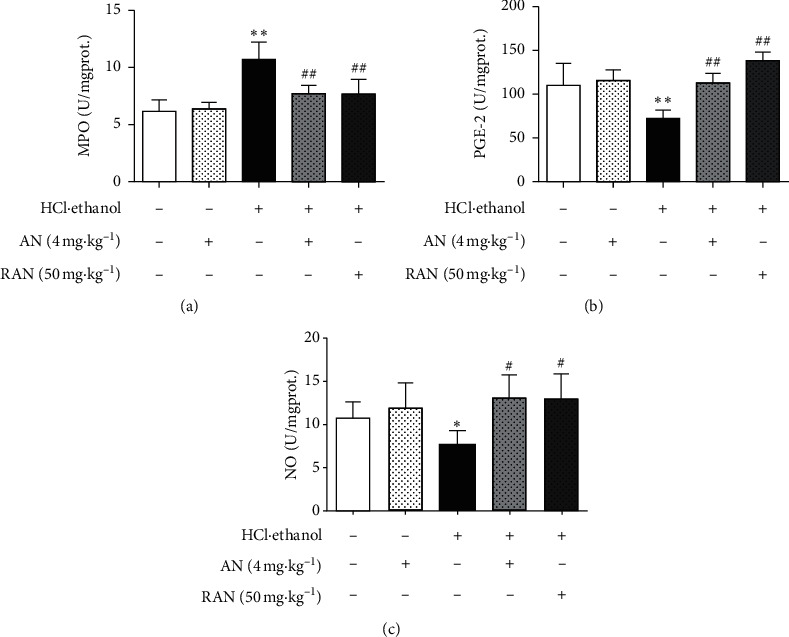
Effects of AN on the contents of MPO, NO, and PGE in the gastric tissues of mice treated with HCl/ethanol. (a) MPO activity. (b) PGE2 content. (c) NO content. The data are presented as mean ± SD (*n* = 8). ^∗^Compared with CON group, *P* < 0.05; ^∗∗^compared with CON group, *P* < 0.01; ^#^compared with HCl/ethanol group, *P* < 0.05; ^##^compared with HCl/ethanol group, *P* < 0.01.

The activity of MPO is directly proportional to the degree of neutrophil infiltration in the tissues, indirectly reflecting the inflammation degree of the tissues [29, 30]. PGE2 and NO are important gastric mucosal protective factors, which play an important role in the body's mucosal defense mechanism [31]. As shown in Figure 7, compared with that in the CON group, MPO content in the gastric tissue of mice in the HCl/ethanol group was significantly increased (*P* < 0.01), and NO and PGE2 contents were significantly decreased (*P* < 0.05 or *P* < 0.01); compared with that in HCl/ethanol group, MPO content in the gastric tissue of mice was significantly decreased (*P* < 0.01), and NO and PGE2 contents were significantly increased (*P* < 0.05 or *P* < 0.01) in HCl/ethanol + AN and HCl/ethanol + RAN groups, while there was no significant difference in the content of these inflammation-related factors in the gastric tissue of mice between CON group and CON + AN group.

These results suggest that AN can reduce the contents of proinflammatory factors TNF-*α*, IL-6, and IL-1*β*; increase the contents of anti-inflammatory factors IL-2, IL-4, and IL-10 and protective factors NO and PGE2 in the gastric tissue of mice treated with HCl/ethanol; and eventually play an anti-inflammatory role and attenuate the gastric ulcer injury in mice.

### 3.6. Upregulation of AN on the Expression of NRF2/ARE Pathway-Related Proteins in the Gastric Tissue of Mice Treated with HCl/Ethanol

**Figure 8:**
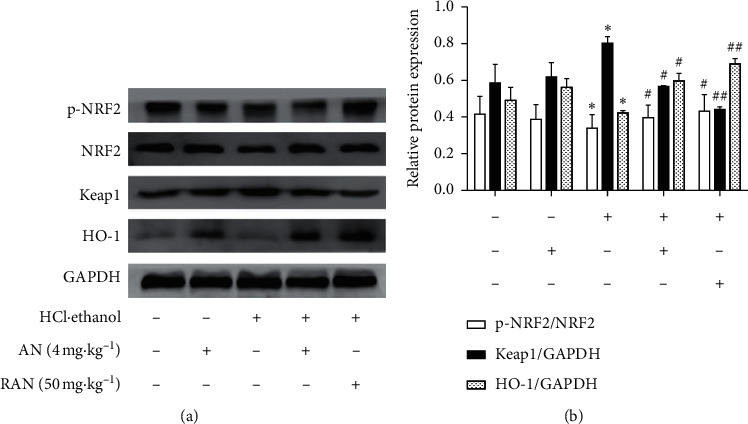
Expressions of NRF2/ARE signaling pathway relative proteins in the gastric tissue of mice. (a) Electrophoretogram of nuclear factor-E2-related factor 2 (NRF2), p-NRF2, Keap1, and HO-1 proteins. (b) Relative expressions of p-NRF2, NRF2, Keap1, and HO-1 proteins. The data are presented as mean ± SD (*n* = 3). ^∗^Compared with CON group, *P* < 0.05; ^∗∗^compared with CON group, *P* < 0.01; ^#^compared with HCl/ethanol group, *P* < 0.05; ^##^compared with HCl/ethanol group, *P* < 0.01.

NRF2/ARE signaling pathway is an important regulatory pathway involved in the body's antioxidant response [32]. As shown in Figure 8, the expression level of Keap1 protein of the gastric tissue of mice in the HCl/ethanol group was significantly higher than that in the CON group (*P* < 0.05), and the expression levels of p-NRF2/NRF2 and HO-1 proteins were significantly lower than those in the CON group (*P* < 0.05 or *P* < 0.01). The expression level of Keap1 protein of the gastric tissue of mice in the HCl/ethanol + AN or HCl/ethanol + RAN group was significantly lower than that in the HCl/ethanol group (*P* < 0.05 or *P* < 0.01). The expression levels of p-NRF2/NRF2 and HO-1 proteins in the gastric tissue of the mice in the HCl/ethanol + AN or HCl/ethanol + RAN group were significantly higher than those in the HCl/ethanol group (*P* < 0.05 or *P* < 0.01), and there was no significant difference in the expression of the proteins between the CON group and CON + AN group. These results suggest that AN may play an antioxidant role by activating the NRF2/ARE signaling pathway and then alleviate the gastric ulcer induced by HCl/ethanol in mice.

### 3.7. Regulation of AN on the Expression of MAPK/NF-*κ*B Pathway-Related Proteins in the Gastric Tissue of Mice Treated with HCl/Ethanol

**Figure 9:**
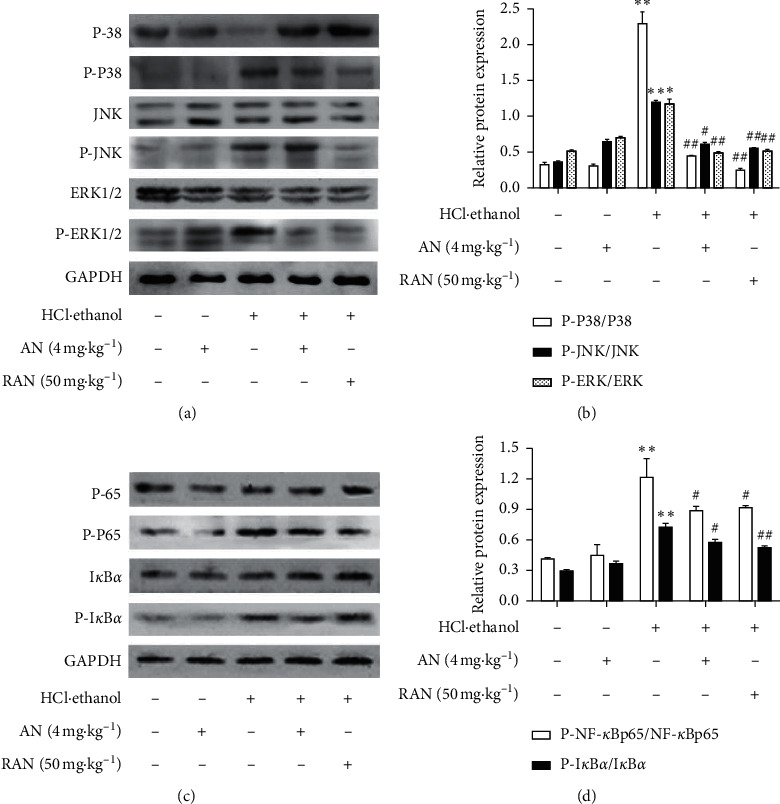
Expression of MAPK/NF-*κ*B signaling pathway related proteins in the gastric tissue of mice treated with HCl/ethanol. (a) Phosphorylated P-38, P-38, phosphorylated JNK, JNK, phosphorylated ERK1/2, and ERK1/2 protein levels. (b) Relative expressions of P-P38/P-38, P-JNK/JNK, and P-ERK/ERK. (c) Phosphorylated nuclear kappa-B (p-NF-*κ*B) p65, nuclear kappa-B (NF-*κ*B) p65, phosphorylated nuclear factor inhibitory protein *α* (p-I*κ*B*α*), and I*κ*B*α* protein levels. (d) Relative expressions of p-NF-kB p65/NF-kB p65 and P-I*κ*B*α*/I*κ*B*α*. The data are presented as mean ± SD (*n* = 3). ^∗^Compared with CON group, *P* < 0.05; ^∗∗^compared with CON group, *P* < 0.01; ^#^compared with HCl/ethanol group, *P* < 0.05; ^##^compared with HCl/ethanol group, *P* < 0.01.

MAPK/NF-*κ*B signaling pathway plays an important regulatory role in the inflammatory response. In this study, the expression levels of proteins related to the MAPK/NF-*κ*B signaling pathway were detected, and the results (Figure 9) showed that, compared with those in the CON group, the ratios of P-P38/p38, P-JNK/JNK, P-ERK1/2/ERK1/2, P-P65/p65, and P-I*κ*B*α*/I*κ*B*α* in the gastric tissue of mice in HCl/ethanol group were significantly increased (*P* < 0.05 or *P* < 0.01), while the pretreatment with AN or RAN could significantly reduce the phosphorylation level of the above proteins (*P* < 0.05 or *P* < 0.01). These results suggest that AN may play an anti-inflammatory role by regulating the MAPK/NF-*κ*B signaling pathway and then alleviate the gastric ulcer induced by HCl/ethanol in mice.

## 4. Discussion

Looking for drugs and health care foods for gastric ulcer is a hot spot and difficult point in medical research. Accordingly, in this study, we chose AN as a candidate drug to study its protective effect on gastric ulcer. Interestingly, the present results show that AN can alleviate the gastric ulcer injuries of mice induced by HCl/ethanol. In brief, AN can reduce the gastric ulcer index, increase the ulcer inhibition rate and improve the pathological damage of gastric tissue of mice. Besides these clinical effects, we further examined the effects of AN on some biochemical markers related to oxidation and inflammation.

Under normal physiological conditions, there is an integrated oxidation-antioxidation balance system in the body [33]. The continuous production and elimination of ROS maintain the dynamic balance of oxidation-antioxidation system, and the imbalance of this system is one of the important reasons for the formation of gastric ulcer [34]. This imbalance usually manifested as the decrease of antioxidant enzymes (such as SOD, CAT, and GSH-Px) [35] and the increase of oxidative stress products (such as MDA and 8-OHdG) [36, 37]. SOD is the most important antioxidant enzyme in the body, maintaining the balance of oxidation and antioxidation [38]. SOD can convert harmful superoxides produced by mitochondrial metabolism into H_2_O_2_ and O_2_, and then H_2_O_2_ is converted to harmless H_2_O and O_2_ under the catalysis of CAT and GSH-Px [39]. On the other hand, excessive ROS can also cause the peroxidation of lipids, proteins, nucleic acid, and other cellular components. Peroxidation, marked by the increase of MDA level, can severely damage the mucosal surface of gastric tissue, eventually resulting in tissue and organ injuries and diseases [40, 41]. At the same time, the accumulation of free radicals can also cause the damage of DNA in cells, and 8-OHdG is the most commonly used biomarker of DNA oxidative damage [42]. In this study, the activities of antioxidant enzymes SOD, CAT, and GSH-Px were significantly decreased, and the contents of ROS, MDA, and 8-OHdG were significantly increased in the gastric tissue; these were consistent with the other reports [43, 44], while the pretreatment with AN could significantly increase the activity of SOD, CAT, and GSH-Px and decrease the content of ROS, MDA, and 8-OHdG. All these suggest that AN may alleviate the gastric ulcer by its antioxidation.

NRF2/ARE signaling pathway is an important regulatory pathway of antioxidant response [45]. NRF2 is one of the major intracellular transcription factors against oxidative stress, and it binds to its negative regulator Keap1 in cytoplasm. When the body suffers oxidative stress, NRF2 dissociates from Keap1 through phosphorylation and binds to the antioxidation element ARE, inducing HO-1 expression [46]. The present results showed that AN could downregulate the expression of Keap1 and upregulate the expressions of p-NRF2/NRF2 and HO-1, indicating that the antioxidant effect of AN may be mediated by activating the NRF2/ARE signaling pathway. Our previous work also showed that AN could activate the NRF2/ARE pathway in the muscle [19], liver, brain, and spleen [20] of mice treated with D-galactose [47], further confirming the correlation between the effect of AN and NRF2/ARE signaling pathway. However, the specific mechanism needs to be further explored.

Inflammation is another important mechanism for gastric mucosal injury induced by HCl/ethanol in mice [48]. The oxidative stress injury in gastric tissue can promote the aggregation and infiltration of neutrophils into the gastric mucosa and regulate the transcription and synthesis of several proinflammatory cytokines, such as TNF-*α*, IL-6, and IL-1*β*, and anti-inflammatory factors, such as IL-2, IL-4, and IL-10 [49]. TNF-*α* can cause an accumulation of a large number of neutrophils around the ulcer, resulting in gastric microcirculation disturbance and the formation of gastric mucosal ulcer [50]. Meanwhile, the high concentration of TNF-*α* can also promote the secretion of cytokines, such as IL-6 and IL-1*β*, leading to inflammation [51]. Anti-inflammatory factors such as IL-2, IL-4, and IL-10 can antagonize TNF-*α*, IL-6, and IL-1*β* to alleviate the inflammatory response [52]. MPO is considered to be a marker of neutrophil infiltration, and the decrease of MPO activity can be interpreted as the enhancement of anti-inflammatory activity in experimental models [53]. The results showed that the pretreatment with AN could significantly lower the content of TNF-*α*, IL-6, IL-1*β,* and MPO in the serum and gastric tissue and increase the content of IL-2, IL-4, and IL-10 at the same time.

Studies have shown that NF-*κ*B, a key transcription factor connecting oxidative stress with inflammatory response, can promote the production of inflammatory factors in the body [54, 55], of which I*κ*B*α* is the inhibitor of NF-*κ*B and P-65 in an inactive state can bind to I*κ*B protein to form P65-P50-I*κ*B*α* complex existing in the cytoplasm in an inactive state. When stimulated by external factors, the NF-*κ*B signaling pathway is activated, then I*κ*B*α* is dissociated from the complex, and P50 and P65 enter the nucleus to regulate the transcription of target genes [56]. Therefore, P65 and I*κ*B*α* are important indicators for the detection of NF-*κ*B signaling pathway [57]. The results of this study showed that HCl/ethanol treatment could significantly increase the phosphorylation level of P65 and I*κ*B*α* in the gastric tissue of mice, which was consistent with the other report [58]. However, after the pretreatment with AN, the phosphorylation levels of P65 and I*κ*B*α* decreased significantly, suggesting that AN could activate NF-*κ*B and then motivate the changes of inflammatory cytokines, thus alleviating the inflammatory reaction in the gastric tissue of mice with the gastric ulcer induced by HCl/ethanol.

The upstream kinase of NF-*κ*B is mainly mitogen-activated protein kinase (MAPK), and the three major subtypes of MAPK family P-38, JNK, and ERK1/2 can play their biological roles only through their phosphorylation and activation [59, 60]. Once activated by kinases, MAPK can phosphorylate the transcription factors or other downstream kinases that regulate the expression of proinflammatory mediators [61]. In this study, we found that AN could significantly reduce the phosphorylation of ERK1/2 of P-38 and JNK, indicating that the anti-inflammatory effect of AN may be related to the inhibition of MAPK/NF-*κ*B signaling pathway to reduce the level of proinflammatory cytokines and promote the release of anti-inflammatory cytokines at the same time. In conclusion, in the process of HCl/ethanol-induced gastric ulcer in mice, ROS mediates the activation of MAPK signaling pathway in the gastric tissue of mice firstly, and then the activation of MAPK further activates the expressions of its downstream transcription factors such as NRF2 and NF-*κ*B; ultimately, these factors improve the antioxidant capacity of gastric tissue to suppress the inflammatory response.

PGE2 and NO, the important gastric mucosal protective factors [62], can protect gastric mucosa by promoting the secretion of mucus and bicarbonate, maintaining the blood flow and limiting the secretion of gastric acid [63]. In addition, PGE2 can also protect gastric mucosa by stimulating the proliferation of gastric mucosal cells and promote the renewal and repair of the mucosal epithelium [64]. The present results showed that AN could increase the content of NO and PGE2 in the gastric mucosa of mice and then alleviate the gastric mucosa from damage.

## 5. Conclusions

**Figure 10:**
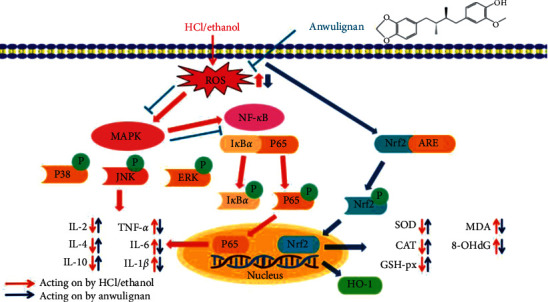
Antiulcer effect of AN through NRF2/ARE and MAPK/NF-*κ*B pathways in mice.

AN has a protective effect against the gastric ulcer induced by HCl/ethanol in mice. As shown in Figure 10, on the one hand, it can enhance the antioxidant capacity of the body by activating the NRF2/ARE signaling pathway and the activity of antioxidant enzymes, such as SOD, CAT, and GSH-Px and, on the other hand, it may play an anti-inflammatory role by inhibiting the release of inflammatory mediators and inducing the release of anti-inflammatory factors through inhibiting the MAPK/NF-*κ*B signaling pathway. Furthermore, AN can increase the levels of PGE2 and NO in the gastric mucosa of mice and then protect the gastric tissue from damage.

## Data Availability

The data used to support the findings of this study are included within the article.
